# Evaluation of the Effectiveness of a Chronic Ocular Hypertension Mouse Model Induced by Intracameral Injection of Cross-Linking Hydrogel

**DOI:** 10.3389/fmed.2021.643402

**Published:** 2021-03-22

**Authors:** Junjue Chen, Jun Sun, Huan Yu, Ping Huang, Yisheng Zhong

**Affiliations:** ^1^Department of Ophthalmology, Ruijin Hospital, Shanghai Jiao Tong University School of Medicine, Shanghai, China; ^2^Shanghai Key Laboratory for Bone and Joint Diseases, Shanghai Institute of Traumatology and Orthopedics, Ruijin Hospital Affiliated Medical School, Shanghai Jiaotong University, Shanghai, China

**Keywords:** glaucoma, animal model, mice, cross-linking hydrogel, intracameral injection

## Abstract

**Background:** Glaucoma is an irreversible and blinding neurodegenerative disease that is characterized by progressive loss of retinal ganglion cells. The current animal models of glaucoma fail to provide a chronic elevated intraocular pressure and cannot maintain the optical media clarity for a long time, which brings some difficulties to the study of glaucoma. Here, we developed a new chronic ocular hypertension model of mice induced by cross-linking hydrogel intracameral injection.

**Methods:** C57BL/6J mice aged 6–8 weeks were randomly divided into the control group and the operation group. The mice of the operation group were injected with cross-linking hydrogel to induce ocular hypertension. Intraocular pressure was measured preoperatively, 3 days after surgery, and weekly until the end of the study. Flash visual evoked potential (F-VEP) was used to observe optic nerve function at different times (preoperatively and 2, 4, and 6 weeks) after chronic ocular hypertension (COH). Retinal TNF-α, IL-1β, and IL-17A protein expression were measured by western blotting in the control group and in mice at 2, 4, and 6 weeks after COH. Microglial cell activation was evaluated by immunofluorescence staining and western blotting. Apoptosis and loss of retinal ganglion cells after 2, 4, and 6 weeks of intracameral injection of cross-linking hydrogel were observed by the TUNEL assay and Brn3a protein labeling. The loss of optic nerve axons in COH mice was evaluated by neurofilament heavy polypeptide protein labeling.

**Results:** Intracameral injection of the cross-linking hydrogel induces increased intraocular pressure (IOP) to a mean value of 19.3 ± 4.1 mmHg, which was sustained for at least 8 weeks. A significant difference in IOP was noted between COH mice and sham-operation mice (*p* < 0.0001). The success rate was 75%. The average amplitude of F-VEP in mice with COH was reduced (*p* = 0.0149, 0.0012, and 0.0009 at 2, 4, and 6 weeks after COH vs. the control group, respectively), and the average latent period in mice with COH was longer (*p* = 0.0290, <0.0001, and <0.0001 at 2, 4, and 6 weeks after COH vs. the control group, respectively) compared with that in the control group. TNF-α, IL-1β, IL-17A, Iba-1, and CD68 protein expression increased in COH mice. During the processing of COH, the number of microglial cells increased along with cellular morphological changes of rounder bodies and thicker processes compared with the control group. Apoptosis of retinal ganglion cells (RGCs) was clearly observed in mice at 2, 4, and 6 weeks after COH (*p* = 0.0061, 0.0012, <0.0001, and 0.0371 at 2, 4, and 6 weeks after COH vs. the control group, respectively). The RGC density decreased significantly in the COH mice compared with the control group (*p* = 0.0042, 0.0036, and <0.0001 at 2, 4, and 6 weeks after COH vs. the control group, respectively). There was a significant loss of optic nerve axons in mice after intracameral injection of cross-linking hydrogel (*p* = 0.0095, 0.0002, and <0.0001 at 2, 4, and 6 weeks after COH vs. the control group, respectively).

**Conclusions:** A single intracameral injection of cross-linking hydrogel can effectively induce chronic ocular hypertension in mice, which causes progressive loss of retinal ganglion cells, increased expression levels of inflammatory cytokines and microglial cell activation, and deterioration of optic nerve function.

## Introduction

Glaucoma, a leading cause of irreversible blindness, is characterized by retinal ganglion cell (RGC) death and axonal damage of the optic nerve (1). Glaucoma affects an estimated 64.3 million people between the ages of 40 and 80 worldwide and is expected to reach 111.8 million by 2040 (2). The pathogenesis of glaucoma has not been elucidated, and the elevated intraocular pressure (IOP) is thought to be a major risk factor (3). However, progressive RGC loss, optic axon injury, and visual field defects are still present in glaucoma patients with normal IOP. This finding suggests that mechanisms other than stress-mediated neurodegenerative injury exist (4). A growing body of literature, particularly from a variety of animal models, suggests that neuroinflammation (often considered to be an immune response associated with the central nervous system) is a key process in glaucoma (5). Retinal microglial cells are one of the main cells involved in immune inflammation in the retina and optic nerve. Microglial cells in the optic nerve head are activated in the early stages of glaucoma. In the case of retinal and optic nerve injury, microglial cells can be rapidly activated to play a beneficial neuroprotective role, but overactivation of microglial cells can lead to damage of the nerve tissue by releasing a series of toxic substances. Simultaneously, activated microglia can deliver antigens to activated T cells in the retina and optic nerve and participate in T-cell-mediated neuroprotective immunity and immunopathological damage (6, 7). However, the specific roles of neuroinflammation and microglia in the development of glaucoma have not been completely elucidated.

Therefore, effective animal models are needed for further study of glaucoma. To date, there are some established animal models of glaucoma, including spontaneous ocular hypertension animal models and induced ocular hypertension animal models. Among the animal models of hereditary glaucoma, the DBA/2J mouse is a commonly used animal model for glaucoma research. However, this model of persistent injury develops slowly, and severe injuries are typically observed at 9 months of age (8). The animal models of induced ocular hypertension include the microbead occlusion glaucoma model, the laser-induced glaucoma model, the optic nerve axotomy model, and the scleral cauterization glaucoma model (9–11). An ideal model of experimental glaucoma should be able to preserve optical media clarity and exhibit chronic, progressive loss of RGCs (12). In addition, the operation is simple, and the cost is reasonable. However, many of the existing models of experimental glaucoma fall short of demonstrating these attributes.

In this study, we used a new method to establish a model of experimental glaucoma—a single cross-linked hydrogel intracameral injection—to induce elevated IOP in mice. The increase in IOP in this model was stable and sustained for at least 8 weeks, leading to the loss of RGCs, increased expression levels of inflammatory cytokines and microglial cell activation, and deterioration of optic nerve function in mice. Our model has the characteristics of strong operability.

## Materials and Methods

### Animals

Six- to 8-week-old male C57BL/6J mice (Charles River Laboratories, Shanghai, China) were used in this study. All animal experimental protocols were approved by Ruijin Hospital, Shanghai Jiao Tong University School of Medicine Animal Care and Use Committee. All animal procedures in this study adhered to the National Institutes of Health (NIH) guidelines for the Care and Use of Laboratory Animals and the Association for Research in Vision and Ophthalmology (ARVO) statement for the Use of Animals in Ophthalmic and Vision Research. All mice were fed *ad libitum*, and the environment was maintained at approximately 21°C with a 12-h light and 12-h dark cycle. The number of animals used in the experimental procedures is listed in Table 1.

**Table 1:** Number of mice used in separate procedures.

**Procedure**	***N* (Mice)**
Intracameral injections for model building and IOP profile	90
Immunofluorescence staining	54
VEP test	24
Western blot	20

### Surgical Induction of the Glaucoma Model

**Figure 1:**
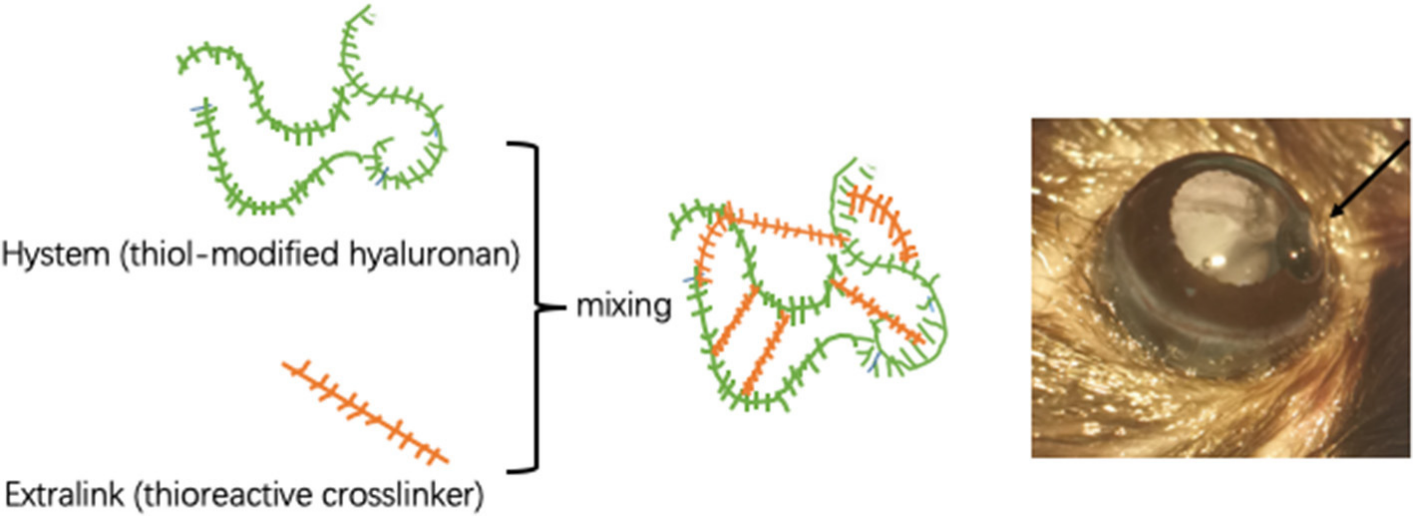
Intracameral injection of cross-linking hydrogel. Schematic diagram of cross-linking hydrogels. The proportion of HyStem and Extralink is 4:1. The hydrogel is injected into the anterior chamber to form solidified gel droplets at the anterior chamber angle.

Chronic ocular hypertension (COH) was induced in the right eyes of mice. To avoid potential inflammatory reactions caused by contralateral COH eyes, the left eye was not considered the control eye (13). Mice were anesthetized by intraperitoneal administration of 80 mg/kg ketamine hydrochloride (Sigma-Aldrich, St. Louis, MO, USA) and 16 mg/kg xylazine (Sigma-Aldrich, St. Louis, MO, USA). Topical anesthesia was delivered to the ocular surface by a drop of 0.5% proparacaine hydrochloride (Bausch & Lomb, Tampa, FL, USA). Before injection, an *in situ* cross-linking hydrogel (Sigma-Aldrich, St. Louis, MO, USA) was mixed. The cross-linking hydrogel consisted of a thiol-modified carboxymethyl hyaluronic acid (HyStem) and a thiol-reactive polyethylene glycol diacrylate (Extralink). Both substances were dissolved in degassed water according to the manufacturer's instructions and shaken in a 37°C thermostat water bath for 2 h before mixing. Anterior chamber puncture is performed from the peripheral area of the cornea to the center with a 31-gauge needle to form a sufficiently long tunnel incision. Then, the premixed hydrogel was immediately injected into the anterior chamber through the incision with a Hamilton syringe (Hamilton Bonaduz AG, Switzerland). The cross-linking hydrogel was mixed at a ratio of 4:1 immediately before the injection and occurred *in situ* for approximately 5 min in the anterior chamber. A total of 3 μl of the mixture including 2.4 μl HyStem and 0.6 μl Extralink was aspirated by a pulled glass micropipette needle and injected into the anterior chamber targeted at the anterior chamber angle (Figure 1). Sham operations were performed on the right eyes of other mice to be used as the control group. Briefly, an equivalent volume of phosphate buffered saline (PBS) was injected into the anterior chamber, and other procedures were the same as described for COH induction.

IOP measurements were performed as previously described (14). Briefly, IOP was measured using a rebound tonometer (TonoLab, Vantaa, Finland) under brief systemic anesthesia by isoflurane inhalation (2–4%) immediately preoperation and 3 days postoperation and weekly until the end point of the experimental period. All IOP measurements were obtained between 10 a.m. and 2 p.m., and the average of six readings was calculated for each IOP measurement.

### Quantification of RGCs and Microglial Cells in Retinal Whole Mounts

Mice were anesthetized and perfused in sequence with saline and 4% paraformaldehyde (PFA) transcardially. The right eyes were enucleated and further fixed in 4% PFA for 1 h at 4°C. Whole retinas were isolated and placed in cold 0.3% Triton X-100 for 30 min and blocked in 10% donkey serum (Jackson ImmunoResearch Laboratories, West Grove, PA, USA) containing 1% bovine serum albumin and 0.05% Tween-20 (Sigma-Aldrich, St. Louis, MO, USA) at room temperature for 1 h. The whole retinas were incubated with rabbit polyclonal anti-Brn3a (1:100 dilution, ab53025; Abcam, Cambridge, UK) primary antibodies for RGCs and rabbit monoclonal anti-Iba-1 (1:500 dilution, ab178846; Abcam, Cambridge, UK) primary antibodies for microglial cells at 4°C overnight. Visualization of immunoreactive proteins was enabled by incubation with goat anti-rabbit secondary antibody (1:1,000 dilution; Thermo Fisher Scientific, Waltham, MA, USA). Immunofluorescence images were scanned and captured with a confocal laser-scanning microscope (LSM 510 META; Zeiss, Jena, Germany). The number of RGCs and microglial cells was measured in areas of approximately the same distances of 1/6, 3/6, and 5/6 retinal radius from the optic disc in each quadrant (each piece of the retina was calculated four times at each distance, the magnification was ×200). A digital image analysis system is used for automatic analysis and counting (Image-Pro Plus Version 6.0, Media Cybernetics, Silver Spring, MD, USA). The average RGC and microglial cell densities were considered the mean density of RGCs and microglial cells for a certain position in each retina.

### Immunofluorescence Staining

Mice were anesthetized and transcardially perfused with saline and 4% PFA. The right eyes and optic nerves were dissected and further fixed in 4% PFA at 4°C overnight, and then cryoprotection was achieved with gradient concentrations of sucrose solutions (the concentration gradients of sucrose solution were 20, 30, and 40%). The eyecups and optic nerves were embedded in optimum cutting temperature compound (Sakura Finetek, Torrance, CA, USA) and frozen. The retinas were nasotemporally sectioned around the optic disc into slices 12 μm thick using a Leica microtome (CM1950, Wetzlar, Germany) and mounted on gelatin-coated slides. The optic nerve cross-sections (12-μm) were cut with a cryostat (CM1950, Leica, Wetzlar, Germany) and mounted on gelatin-coated slides. Slices were permeated with cold 0.3% Triton X-100 for 30 min and blocked in 10% donkey serum (Jackson ImmunoResearch Laboratories, West Grove, PA, USA) containing 1% bovine serum albumin and 0.05% Tween-20 (Sigma-Aldrich, St. Louis, MO, USA) at room temperature for 1 h. The retinal slices were incubated with rabbit polyclonal anti-Brn3a (1:100 dilution, ab53025; Abcam, Cambridge, UK) primary antibodies, and the optic nerves slices were incubated with rabbit monoclonal anti-neurofilament heavy polypeptide (1:100 dilution, ab207176; Abcam, Cambridge, UK) primary antibodies at 4°C overnight. Visualization of immunoreactive proteins was enabled by incubation with goat anti-rabbit secondary antibody (1:1,000 dilution; Thermo Scientific, Waltham, MA, USA). For RGCs, nuclear DNA fragmentation of apoptotic cells was evaluated using the terminal deoxynucleotidyl transferase-mediated deoxyuridine triphosphate nick end labeling (TUNEL) method, employing an *in situ* Cell Death Detection Kit (Roche Diagnostics Corporation, Indianapolis, IN, USA). The procedures followed the manufacturer's instructions. Briefly, after incubation with goat anti-rabbit secondary antibody, the slices were incubated with 50 μl of TUNEL reaction agent, containing 5 μl of enzyme solution and 45 μl of label solution, in a humidified incubator for 1 h at 37°C. Finally, the nuclear was mounted with a ProLong Antifade medium combined with 4′,6-diamidino-2-phenylindole (DAPI; Life Technologies, Waltham, MA, USA). The fluorescence images were scanned and captured with a confocal laser-scanning microscope (LSM 510 META; Zeiss, Jena, Germany). Quantitative analysis of fluorescence density was performed according to a previous study (14, 15). Briefly, for retinal slices, three randomly selected nonoverlapping subranges of 300 μm within a 1-mm distance from the optic disc margin of each unilateral side were outlined for a total of six subranges for each slice. Then, the mean optical density of immunoreactive fluorescence staining was measured within distinct areas. The numbers of Brn3a-positive cells and double-staining-positive cells within the ganglion cell layer (GCL) were counted; thus, the proportion of TUNEL-positive neurons in the GCL was obtained. Finally, for each staining target, 18 values obtained from one eye were expressed as an average for individual mice. For optic nerves, five sections per slide and three images per optic nerve were captured. Images were converted into gray scales and the background was subtracted. Then, the lower and upper threshold values were determined for each image. Finally, a digital image analysis system was used to calculate the average fluorescence intensity of the selected area. All the image analysis work was performed with the help of a digital image analysis system (Image-Pro Plus Version 6.0, Media Cybernetics, Silver Spring, MD, USA).

### Flash Visual Evoked Potential

The flash visual evoked potential (F-VEP) test (UTAS-E3000LKC, Multi-focal Visual Diagnostic Test System, IKC Technologies, Gaithersburg, MD, USA) was performed in mice preoperatively and 2, 4, and 6 weeks postoperatively. The method was performed according to the standards of the International Clinical Electrophysiology of Vision (16). The silver needle electrode was used with an impedance of 2k. Using a full-field Ganzfeld flash stimulator, the stimulated light intensity was 3.12 cd s^−1^ m^−2^. The magnification was 20,000 times. The low frequency was 0.1 Hz and the high frequency was 300 Hz. The single stimulus mode was adopted, and the stimulus frequency was 1.0 Hz. The analysis time was 250 ms, and the waveform was superposed 100 times. The mice were anesthetized and dilated with tropicamide. The mice were placed on a homemade mouse fixation device. The recording electrode was placed under the occipital tuberosity scalp. The reference electrode was placed under the nose, and the ground electrode was placed under the mastoid process. After the electrodes were placed, the mice underwent dark adaptation for 15 min. When one eye was examined, the opaque black blindfold completely covered the opposite eye. F-VEP inspection was performed using the two-channel recording method and measured at least thrice consecutively.

### Western Blotting Analysis

The COH mice were sacrificed by cervical dislocation, and the retinas were collected immediately and homogenized. Total protein content was extracted from the homogenate using radioimmunoprecipitation assay buffer (RIPA; Sigma-Aldrich, St. Louis, MO, USA) with a protease inhibitor cocktail (Sigma-Aldrich, St. Louis, MO, USA). Retinal tissue (*n* = 2/group) was homogenized in 100 μl RIPA containing a protease inhibitor cocktail on ice for 1 h. Subsequently, all samples were centrifuged at 15,000×*g* at 4°C for 15 min, and the supernatant was applied to determine the protein concentration. The protein concentrations were measured using the Bicinchoninic Acid Kit (Beyotime, Shanghai, China) (the protein concentration was 3 μg/μl). Briefly, 5× SDS buffer was added to each protein sample (4:1) and denaturized at 100°C for 10 min. Equal amounts of protein samples were separated by electrophoresis in a Mini-Protean three electrophoresis system (Bio-Rad, Hercules, CA, USA) with sodium dodecyl sulfate polyacrylamide gel. Then, proteins were electroblotted to polyvinylidene fluoride membranes in a Mini Trans-Blot electrophoretic transfer system (Bio-Rad, Hercules, CA, USA). The membranes were blocked with 5% skimmed milk at room temperature for 2 h and subsequently incubated with rabbit polyclonal anti-actin (1:5,000 dilution; ab179467; Abcam, Cambridge, UK), mouse monoclonal anti-TNF-α (1:1,000 dilution; ab1793; Abcam, Cambridge, UK), rabbit polyclonal anti-IL-1β (1:1,000; ab9722; Abcam, Cambridge, UK), rabbit monoclonal anti-Iba-1 (1:1,000 dilution; ab178846; Abcam, Cambridge, UK), rabbit monoclonal anti-IL-17A (1:1,000 dilution; ab79056; Abcam, Cambridge, UK), and mouse monoclonal anti-CD68 (1:200 dilution; ab201340; Abcam, Cambridge, UK) primary antibodies at 4°C overnight. The membranes were then washed sufficiently with Tris-buffered saline with Tween and incubated with horseradish peroxidase-conjugated goat anti-rabbit (1:1,000 dilution; Abcam, Cambridge, UK) and goat anti-mouse (1:1,000 dilution; Abcam, Cambridge, UK) secondary antibodies at room temperature for 1 h according to the sources of primary antibodies. The protein bands on the membranes were visualized with a chemofluorescence reagent (Beyotime, Shanghai, China) under an ImageQuant LAS 4000 mini system (GE Healthcare Bio-Sciences, Piscataway, NJ, USA). Gray values of the images were analyzed semiquantitatively with Image-Pro Plus Version 6.0 software (Media Cybernetics, Silver Spring, MD, USA).

### Statistical Methods

Statistical analyses were performed using SPSS software (19.0 IBM Corporation, Armonk, NY, USA). First, the normal distribution of the data was tested. Then the data were analyzed using two-tailed independent-samples *t* test and one-way ANOVA followed by Tukey's *post hoc* test. These tests were used to evaluate statistical significance. *p* < 0.05 indicated that the difference was statistically significant.

## Results

### Longitudinal Profile of IOP Elevation

**Figure 2:**
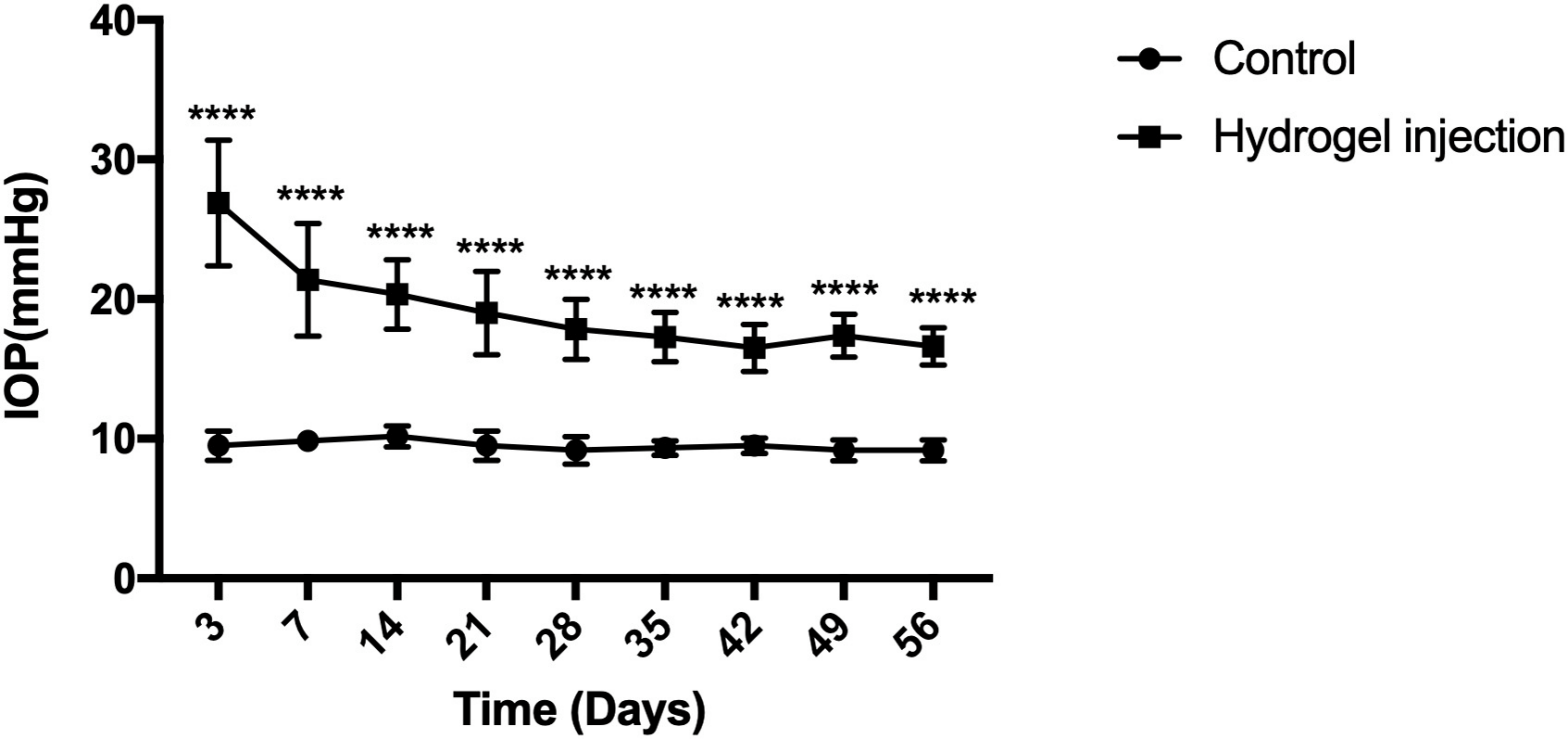
Elevation of intraocular pressure (IOP) after intracameral injection of cross-linking hydrogel. The IOP of hydrogel injection eyes decreased slowly from the first measurement and then processed into a relatively stable phase of ocular hypertension for at least 8 weeks (average, 19.3 ± 4.1 mmHg). The control group presented a stable level of IOP throughout the whole period of the experiment (average, 9.5 ± 0.8 mmHg). There was statistical difference between the two groups (*p* < 0.0001). Data were analyzed using two-tailed independent-samples *t* test followed by Bonferroni test (****p < 0.0001, the group with injection of cross-linking hydrogel vs. the control group). *N* = 24 mice (control group: 6 mice; hydrogel injection group: 18 mice). Bars represent mean ± SD.

Mice were randomly assigned for anterior chamber injection. Fifty-four eyes exhibited elevated IOP after intracameral injection of cross-linking hydrogel and the success rate was 68% (54/80). A total of 80 mice were injected with cross-linking hydrogel. However, eight of them showed postoperative opacity of refractive media, so these eight mice were not counted. The success rate of mice with clear refractive media was 75% (54/72). There were no adverse effects in the anterior chamber after intracameral injection (such as endophthalmitis, keratitis, corneal neovascularization, corneal opacities, incision leakage, cataracts, and ulceration). There were 82 mice which consist of 10 mice of the control group and 72 mice of the COH group during the process of the whole experimental period. In order to reduce the statistical error, 6 mice in the control group and 18 mice in the hydrogel injection group were used for the value of IOP analysis. The preoperative IOP was similar between the hydrogel-injected eyes and the control group (9.5 ± 1.0 and 9.8 ± 0.9 mmHg, *p* = 0.4994). The mean IOP of the hydrogel-injected eyes and the control group was 19.3 ± 4.1 and 9.5 ± 0.8 mmHg, respectively (this value is the average of 240 IOP measurements in the entire experimental period, *p* < 0.0001). In the operation group, IOP decreased slowly from the first measurement, remained relatively stable at the third week after the operation, and was sustained for at least 8 weeks compared with the control group. The difference between the hydrogel-injected eyes and the control group appeared significant at every time point after the intracameral injection (Table 2). The above results suggested that the injection of cross-linking hydrogel can induce IOP elevation successfully (Figure 2).

**Table 2:** Summary of IOP measurements preoperation and postoperation in mice.

**Time**		**Group (*n* = 24)**	**IOP (mmHg)**	**Significance**
			**(mean ± SD)**	**(*p*) vs. control**
Preoperation		Control	9.8 ± 0.9	–
		COH	9.5 ± 1.0	0.4994
Postoperation	Day 3	Control	9.5 ± 1.0	–
		COH	26.9 ± 4.5	<0.0001
	Week 1	Control	9.8 ± 0.8	–
		COH	21.4 ± 4.0	<0.0001
	Week 2	Control	10.1 ± 0.8	–
		COH	20.3 ± 2.5	<0.0001
	Week 3	Control	9.5 ± 1.0	–
		COH	19.0 ± 2.9	<0.0001
	Week 4	Control	9.2 ± 1.0	–
		COH	17.8 ± 2.1	<0.0001
	Week 5	Control	9.3 ± 0.5	–
		COH	17.3 ± 1.8	<0.0001
	Week 6	Control	9.5 ± 0.5	–
		COH	16.5 ± 1.7	<0.0001
	Week 7	Control	9.2 ± 0.8	–
		COH	17.4 ± 1.6	<0.0001
	Week 8	Control	9.2 ± 0.8	–
		COH	16.6 ± 1.3	<0.0001

*The 24 mice include 6 mice of the control group and 18 mice of the COH group. Data were analyzed using two-tailed independent-samples t test followed by Bonferroni test*.

### Flash Visual Evoked Potential

**Figure 3:**
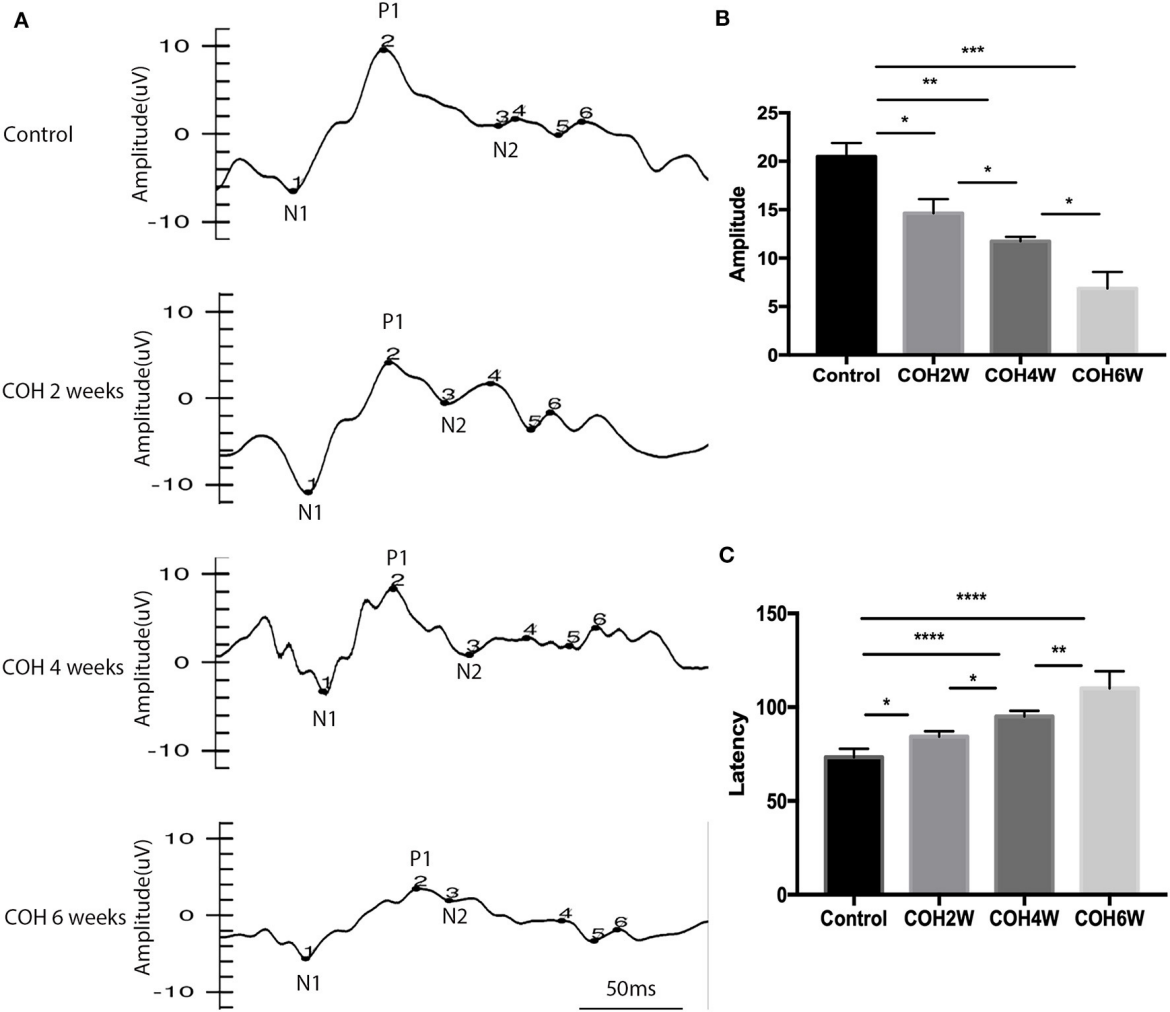
Flash visual evoked potential (F-VEP) test result of C57BL/6J mice. **(A)** The F-VEP test was measured before and 2, 4, and 6 weeks after IOP elevation induced by intracameral injection of hydrogel. **(B)** The amplitudes of P1 were significantly reduced in the hydrogel-injected eyes compared with those in the control group. **(C)** The latent periods of P1 were significantly prolonged in mice at 2, 4, and 6 weeks of COH compared with those in the control group. Data were analyzed using one-way ANOVA followed by Tukey's *post hoc* test (*p < 0.05, **p < 0.01, ***p < 0.001, and ****p < 0.0001). *N* = 24 mice (control group: 6 mice; hydrogel injection group: 18 mice). Bars represent mean ± SD.

F-VEP was examined in mice preoperatively, and mice were subjected to 2, 4, and 6 weeks of COH. The average amplitude of P1 in the control group was 20.5 ± 3 μV, and the average amplitudes of P1 in mice at 2, 4, and 6 weeks of COH were 14.6 ± 1.5, 11.7 ± 0.5, and 6.9 ± 1.7 μV, respectively. There was a significant difference between the eyes after intracameral injection of cross-linking hydrogel and the control group (*p* = 0.0149, 0.0012, and 0.0009 at 2, 4, and 6 weeks after COH vs. the control group, respectively). The average latent period of N1 in the control group was 73.3 ± 4.5 ms, and the latent periods of P1 in mice at 2, 4, and 6 weeks of COH were 84.3 ± 2.9, 95.0 ± 3.0, and 110.0 ± 9.2 ms, respectively. A significant difference in the latent period was noted between the eyes with elevated IOP induced by cross-linking hydrogel and the control group (*p* = 0.0239, <0.0001, and <0.0001 vs. 2, 4, and 6 weeks of COH vs. the control group, respectively) (Figure 3).

### Expression of Inflammatory Factors After Intracameral Injection of Cross-Linking Hydrogel

**Figure 4:**
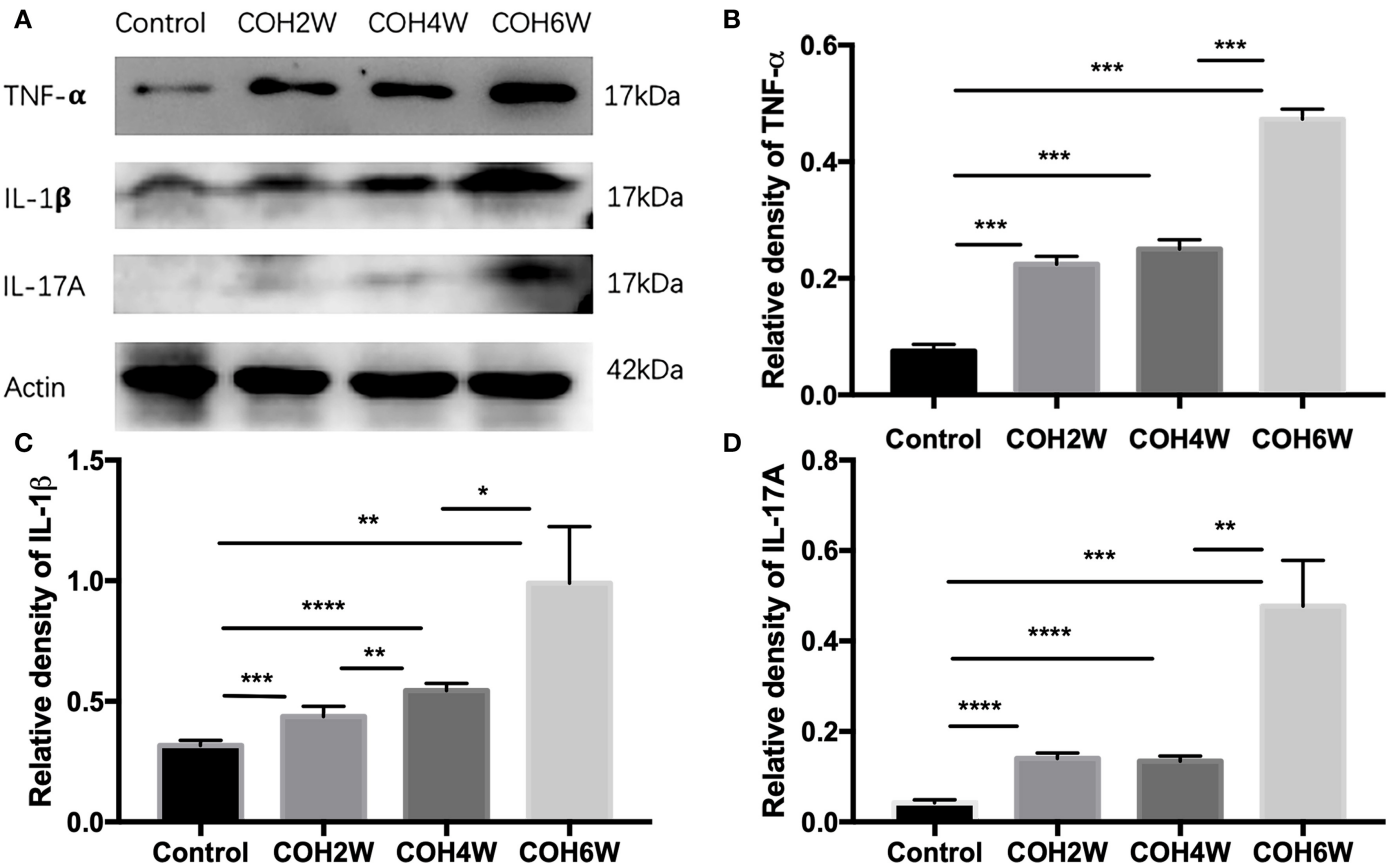
Western blotting of retinal expressions of inflammatory factors. **(A)** Bands of western blotting. **(B**–**D)** Quantitative analysis of western blotting gray values; TNF-α, IL-1β, and IL-17A protein expression increased gradually from the second week to the sixth week after COH. Data were analyzed using one-way ANOVA followed by Tukey's *post hoc* test (*p < 0.05, **p < 0.01, ***p < 0.001, and ****p < 0.0001). *N* = 20 mice (control group: 5 mice; hydrogel injection group: 15 mice). Bars represent mean ± SD.

Western blotting showed that retinal tumor necrosis factor alpha (TNF-α), interleukin (IL)-1β, and IL-17A protein expression in COH mice generally increased over time (Figure 4A). At the second week after COH, TNF-α protein expression was increased compared with the control group by 14.9% (*p* < 0.0001). At the fourth and sixth weeks after COH, TNF-α protein expression increased by 17.5 and 39.7%, respectively (*p* < 0.0001 at each group vs. the control group). At the fourth week after COH, TNF-α protein expression showed no significant difference from the second week after COH (Figure 4B). IL-1β protein expression was increased by 11.9, 22.8, and 67.2% at the second, fourth, and sixth weeks after COH, respectively (*p* = 0.0006, *p* < 0.0001, and 0.0028 vs. the control group, respectively) (Figure 4C). IL-17A protein expression was increased by 9.8, 9.1, and 43.5% at the second, fourth, and sixth weeks after COH, respectively (*p* < 0.0001, <0.0001, and 0.0005 vs. the control group, respectively) (Figure 4D). However, no significant difference in IL-17A protein expression was noted between 2 and 4 weeks after COH.

### Increase in Microglial Cell Activation Under IOP Elevation

**Figure 5:**
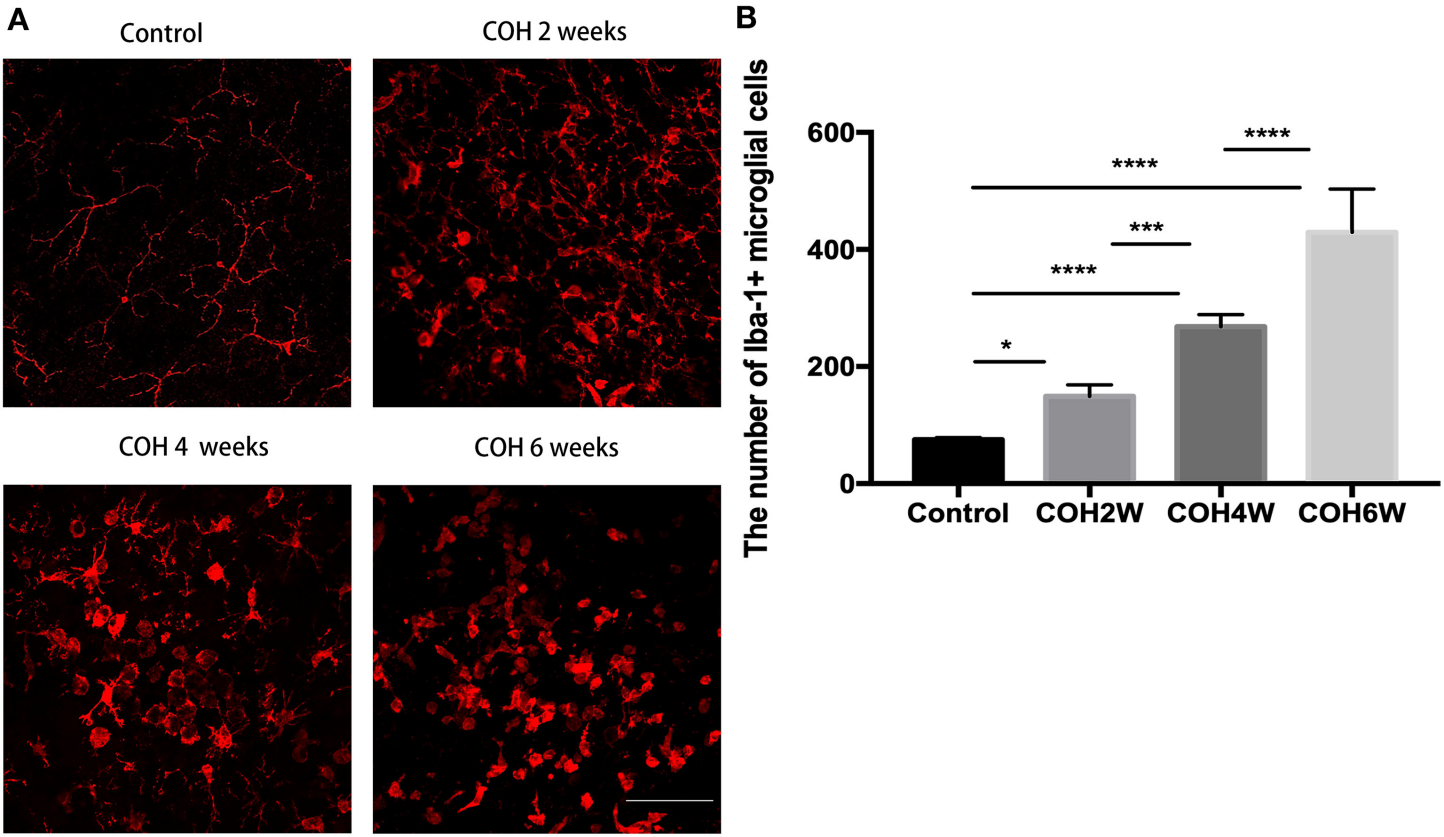
Activation of microglial cells induced by COH. **(A)** Immunofluorescence staining of Iba-1 proteins in retinal whole mounts. In the group of COH, microglial cell showed amoeba morphological change (magnification ×200, scale bar = 50 μm). **(B)** The number of Iba-1+ microglial cells in COH mice. The number and activation of microglial cell increased with the prolonged duration of COH. Data were analyzed using one-way ANOVA followed by Tukey's *post hoc* test (*p < 0.05, ***p < 0.001, and ****p < 0.0001). *N* = 16 mice (control group: 4 mice; hydrogel injection group: 12 mice). Bars represent mean ± SD.

**Figure 6:**
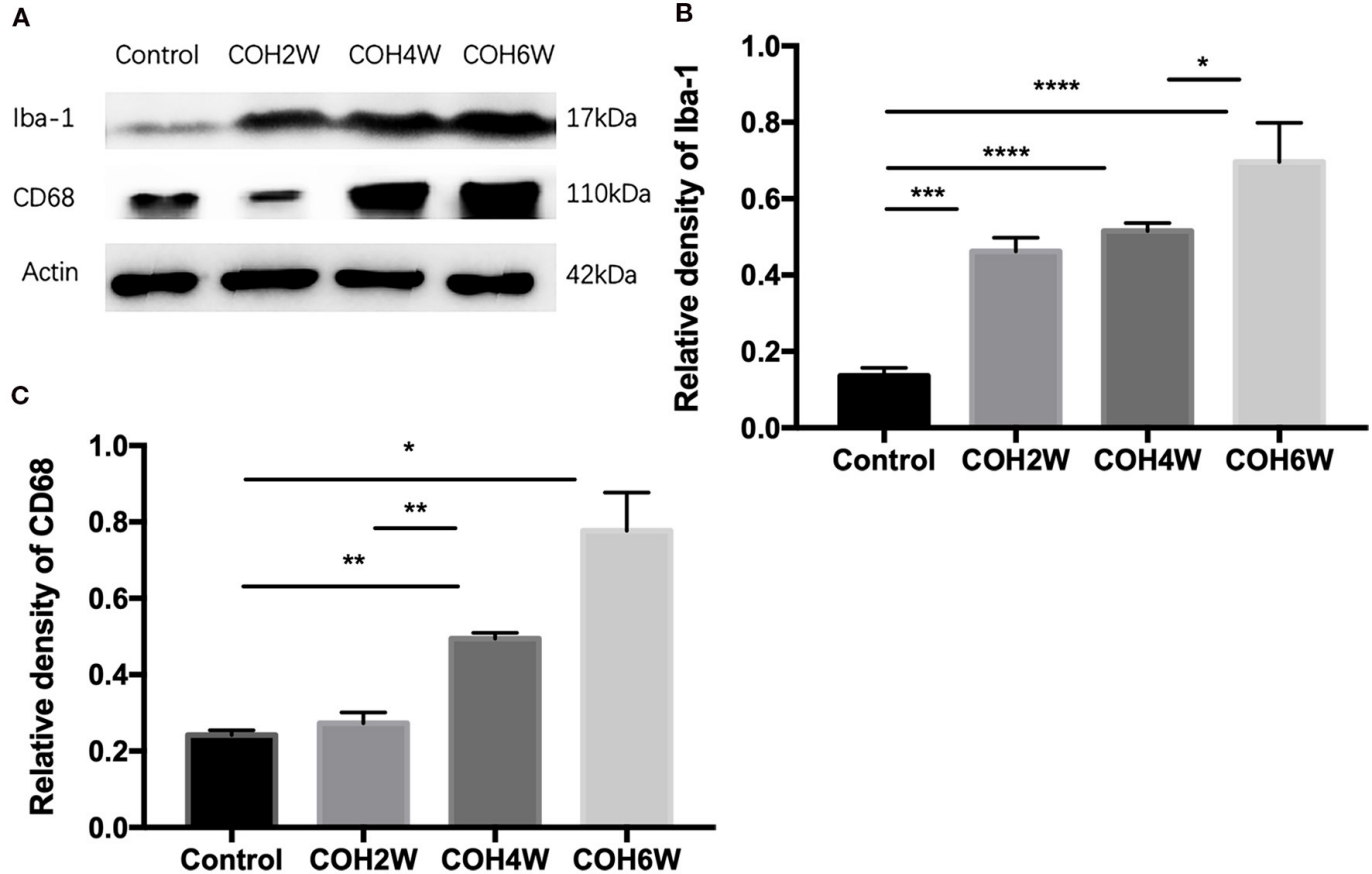
Western blotting of retinal expressions of Iba-1 and CD68. **(A)** Bands of western blotting. **(B,C)** Quantitative analysis of western blotting gray values. The expression of Iba-1 and CD68 increased after the second week of COH. Data were analyzed using one-way ANOVA followed by Tukey's *post hoc* test (*p < 0.05, **p < 0.01, ***p < 0.001, and ****p < 0.0001). *N* = 20 mice (control group: 5 mice; hydrogel injection group: 15 mice). Bars represent mean ± SD.

Immunofluorescent images showed microglial cell activation early in the COH group. The number of Iba-1+ microglial cells in the control group and in mice at 2, 4, and 6 weeks of COH was 75.3 ± 3.5, 149.4 ± 19.3, 268.0 ± 20.7, and 429 ± 74.1/mm^2^, respectively. Compared with the control group, the number of microglial cells in the retina of the COH group was significantly increased (*p* = 0.0258, <0.001, and <0.001 vs. the control group, respectively) (Figure 5B). The cell morphology became rounder, and the processes were thicker (Figure 5A). Western blotting results also demonstrated that retinal Iba-1 and CD68 protein expression levels were both markedly upregulated in the COH group (Figure 6A). Iba-1 protein expression increased by 32.6, 37.9, and 56.0% at the second, fourth, and sixth weeks after COH, respectively. A significant difference was noted between the COH mice and the control group (*p* = 0.0005, 0.0002, and <0.0001, respectively). CD68 protein expression was increased by 3.1, 25.1, and 53.4% at the second, fourth, and sixth weeks after COH, respectively. A significant difference was noted in mice between the fourth and sixth weeks of COH and the control group (*p* = 0.0099 and 0.0356, respectively) (Figures 6B,C).

### Decrease of RGCs After Intracameral Injection of Cross-Linking Hydrogel

**Figure 7:**
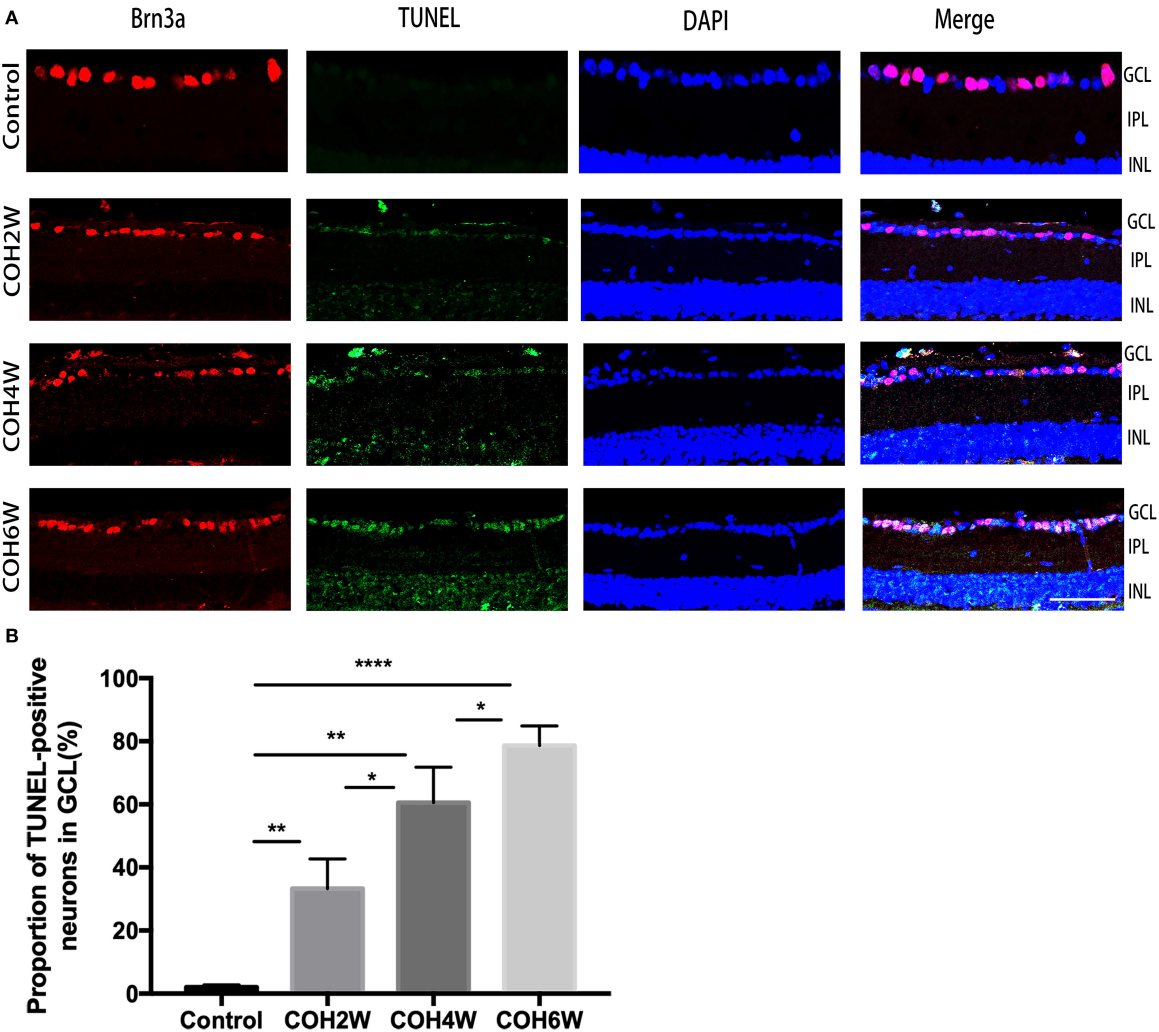
Apoptosis of neurons in the ganglion cell layer (GCL) was obvious after intracameral injection of cross-linking hydrogel. **(A)** Representative images of TUNEL (green fluorescence) and Brn3a (red fluorescence) double-staining for apoptosis evaluation of neurons in the GCL of different groups (magnification ×200, scale bar = 50 μm). Cell nuclei were marked with DAPI (blue). **(B)** Quantitative analysis of proportions of TUNEL-positive cells to Brn3a-positive cells in the GCL (*p < 0.05, **p < 0.01, and ****p < 0.0001). Data were analyzed using one-way ANOVA followed by Tukey's *post hoc* test. *N* = 24 mice (control group: 6 mice; hydrogel injection group: 18 mice). Bars represent mean ± SD.

The double label of Brn3a and the TUNEL assay were used to evaluate the apoptosis of RGCs in the retinas of mice after intracameral injection of cross-linking hydrogel. The TUNEL assay showed that RGC apoptosis was obvious in mice after 2, 4, and 6 weeks of COH (Figure 7A). The proportion of TUNEL-positive neurons in the GCL was 2.0 ± 0.8, 33.3 ± 9.5, 60.6 ± 11.3, and 78.6 ± 6.3% of the control group and of mice after 2, 4, and 6 weeks of COH, respectively (*p* = 0.0061, 0.0012, <0.0001, and 0.0371 vs. the control group, respectively) (Figure 7B), indicating that elevated IOP induced by cross-linking hydrogel injection can lead to RGC apoptosis.

**Figure 8:**
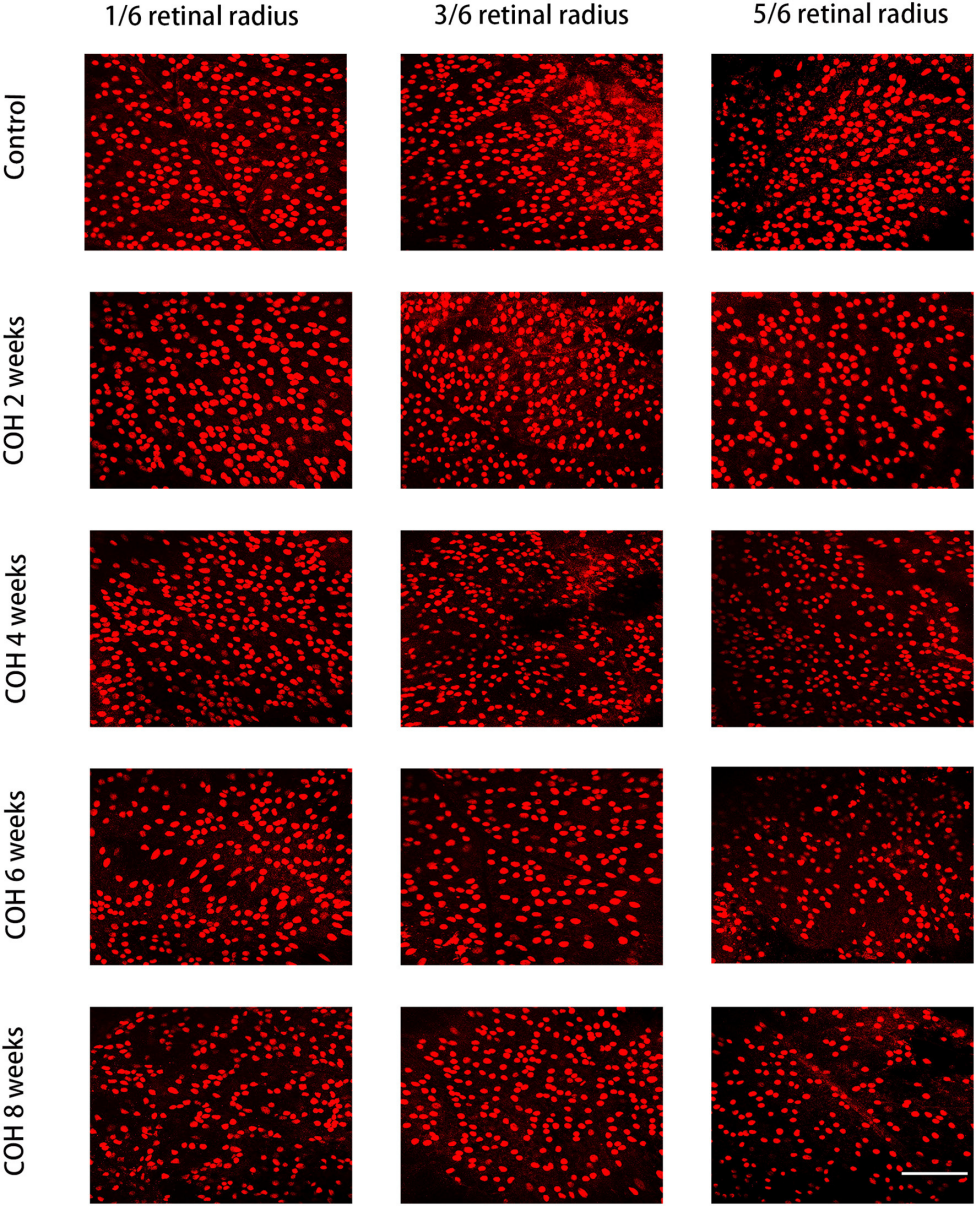
Density of retinal ganglion cells (RGCs) in the control group and COH mice. The label of RGCs with Brn3a in 1/6, 3/6, and 5/6 retinal radius in mice at 2, 4, 6, and 8 weeks of COH (magnification ×200, scale bar = 50μm). *N* = 12 mice (control group: 3 mice; hydrogel injection group: 9 mice).

**Figure 9:**
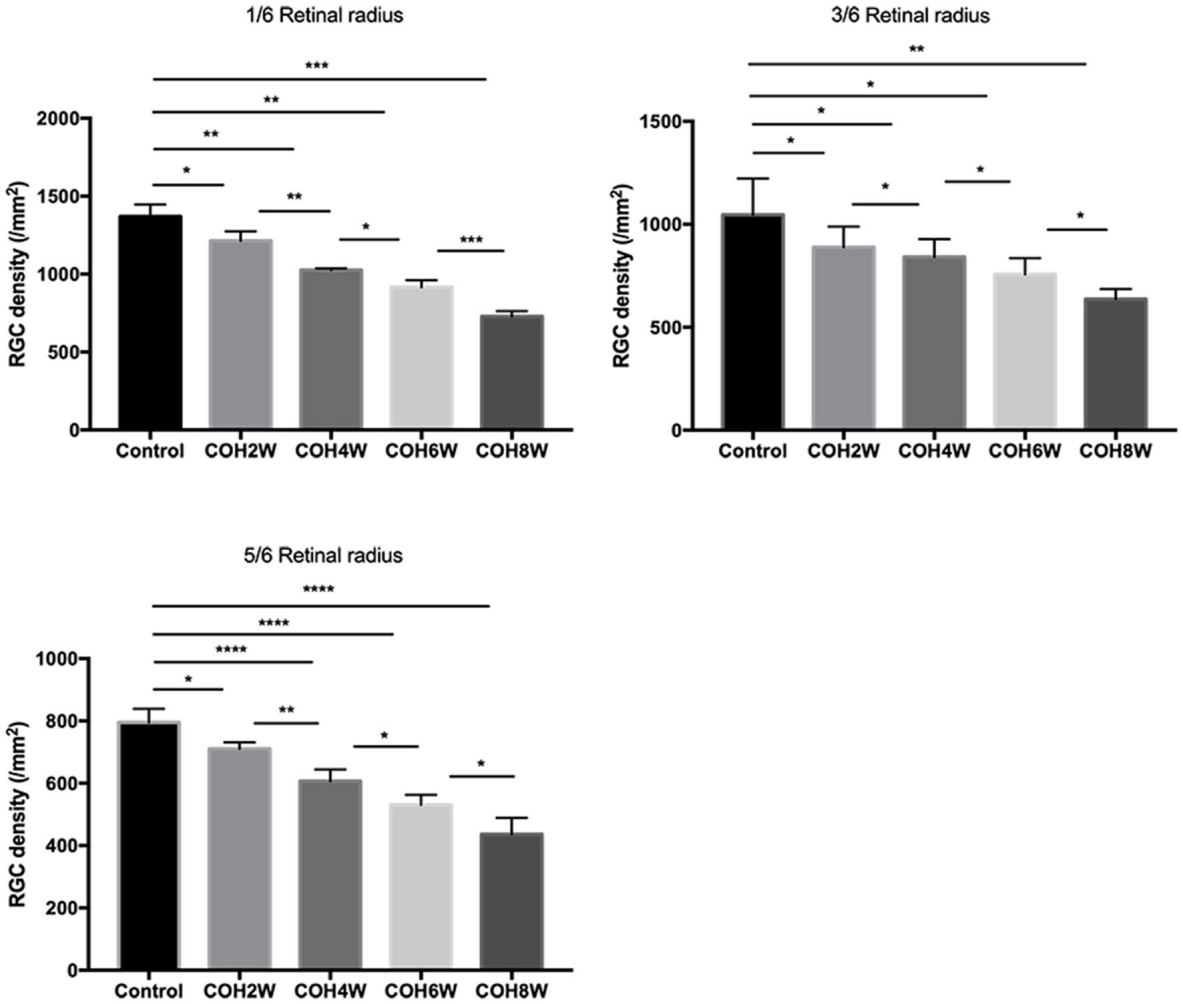
Quantitative analysis of surviving RGCs after COH. Two, 4, 6, and 8 weeks following the induction of COH, the RGC density in 1/6, 3/6, and 5/6 retinal radius was significantly lower than that in the control group. Data were analyzed using one-way ANOVA followed by Tukey's *post hoc* test (*p < 0.05, **p < 0.01, ***p < 0.001, and ****p < 0.0001). *N* = 12 mice (control group: 3 mice; hydrogel injection group: 9 mice). Bars represent mean ± SD.

Immunofluorescence staining with Brn3a in retinal whole mounts was performed in mice 2, 4, 6, and 8 weeks following intracameral injection of the hydrogel and in the control group. In the control group, the number of RGCs was 1,369.0 ± 77.4/mm^2^ in 1/6 retinal radius, 1,046.0 ± 175.3/mm^2^ in 3/6 retinal radius, and 795.0 ± 43.8/mm^2^ in 5/6 retinal radius, respectively. In the COH group, the RGC density decreased significantly in all three retinal positions compared with the control group (Figure 8). The number of RGCs in the COH 2-week group (1,213.0 ± 61.4/mm^2^ in 1/6 retinal radius, 888.2 ± 100.6/mm^2^ in 3/6 retinal radius, and 710.6 ± 21.1/mm^2^ in 5/6 retinal radius, respectively) decreased markedly in comparison with the control group (*p* = 0.0366, 0.0495, and 0.0181, respectively). The number of RGCs in the COH 4-week group (1,027.0 ± 10.5/mm^2^ in 1/6 retinal radius, 841 ± 86.9/mm^2^ in 3/6 retinal radius, and 606.6 ± 38.0/mm^2^ in 5/6 retinal radius, respectively), the COH 6-week group (916.6 ± 43.4/mm^2^ in 1/6 retinal radius, 756.6 ± 78.7/mm^2^ in 3/6 retinal radius, and 530.8 ± 32.5/mm^2^ in 5/6 retinal radius, respectively), and the COH 8-week group (728.6 ± 33.5/mm^2^ in 1/6 retinal radius, 636.6 ± 49.7/mm^2^ in 3/6 retinal radius, and 436.5 ± 52.5/mm^2^ in 5/6 retinal radius, respectively) further decreased in comparison with the control group, respectively (COH 4 weeks, *p* = 0.0037, 0.0310, and <0.0001 vs. the control group, respectively; COH 6 weeks, *p* = 0.0037, 0.0187, and <0.0001 vs. the control group, respectively; COH 8 weeks, *p* = 0.0038, 0.0187, and <0.0001 vs. the control group, respectively). In mice at 8 weeks of COH, the rates of RGC loss were 46.8% in 1/6 retinal radius, 39.2% in 3/6 retinal radius, and 45.1% in 5/6 retinal radius, respectively (Figure 9).

### Optic Nerve Axon Loss After Intracameral Injection of Cross-Linking Hydrogel

**Figure 10:**
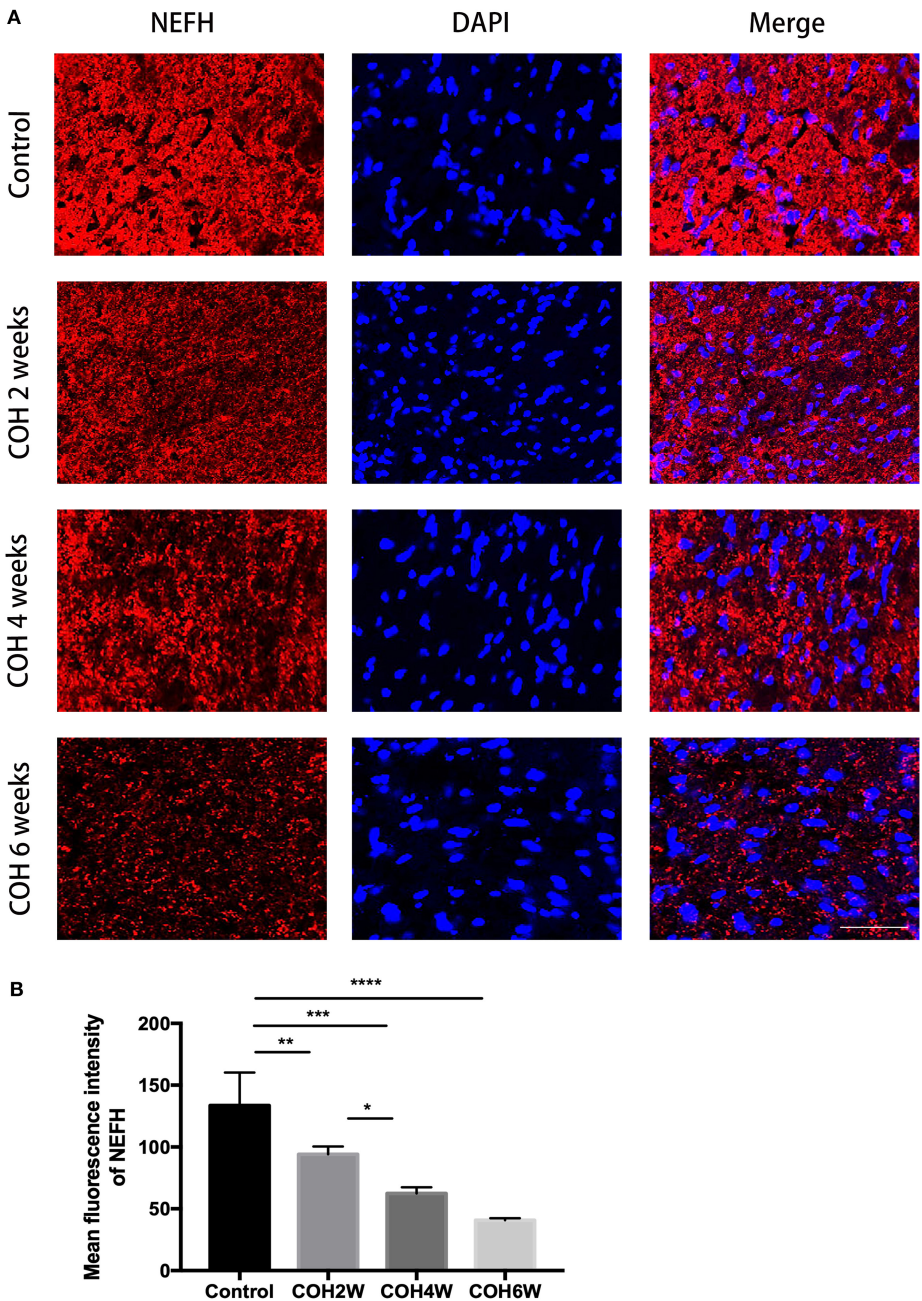
Quantitative analysis of axon loss after COH. **(A)** Optic nerve slices were stained with NEFH (red fluorescence) and cell nuclei were marked with DAPI (blue fluorescence) (magnification ×200, scale bar = 50 μm). **(B)** Quantitative analysis of mean fluorescence intensity of NEFH in the control group and the COH mice. COH mice showed a significant decrease of NEFH compared with the control group. Data were analyzed using one-way ANOVA followed by Tukey's *post hoc* test (*p < 0.05, **p < 0.01, ***p < 0.001, and ****p < 0.0001). *N* = 12 mice (control group: 3 mice; hydrogel injection group: 9 mice). Bars represent mean ± SD. NEFH, neurofilament heavy polypeptide.

To analyze the loss of optic nerve axons in mice with intracameral injection of cross-linking hydrogel, immunofluorescence staining with neurofilament heavy polypeptide (NEFH) was performed in optic nerve cross-sections of the control group and COH mice. The mean fluorescence intensity of NEFH in the control mice was 133.6 ± 26.7. The mean fluorescence intensity (MFI) of NEFH was significantly reduced in mice at 2, 4, and 6 weeks of COH compared with that of the control group (*p* = 0.0095, 0.0002, and <0.0001 vs. the control group, respectively) (Figure 10A). The MFI of NEFH in mice at 2 weeks of COH was 93.9 ± 6.3; in mice at 4 weeks of COH, it was 62.4 ± 5.0; and in mice at 6 weeks of COH, it was 40.7 ± 1.7 (Figure 10B). The rate of optic nerve axon loss was 69.5% at 6 weeks of COH.

## Discussion

Glaucoma is a group of diseases characterized by the loss of the visual field and progressive damage to RGCs and axons, which eventually lead to irreversible blindness (1). The pathological core is the chronic neurodegeneration. At present, the mechanism of glaucoma optic nerve degeneration is not completely understood. Glaucoma is considered to be an optic neurodegenerative disease caused by multiple factors, including mechanical damage of elevated IOP, neurotrophic factor deprivation, ischemia or reperfusion injury, oxidative stress injury, excitatory glutamate toxicity, and abnormal immune inflammatory response (17–19). Therefore, it is very important to construct an effective animal model of ocular hypertension for future glaucoma research.

In our study, we used a cross-linking hydrogel for intracameral injection to induce COH in mice and to study chronic degeneration of the RGCs and visual function in experimental glaucoma. Intracameral injection of cross-linking hydrogel effectively increased the IOP in mice for at least 8 weeks. In the operation group, IOP decreased slowly from the first measurement and remained relatively stable at the third week after the operation. The postoperative mean IOP was 19.3 ± 4.1 mmHg. The IOP of the cross-linked hydrogel-injected eyes was significantly increased compared with that of the control group. Immunofluorescent images and western blotting showed that the expression of inflammatory mediators, such as IL-17A, was increased, and microglial cells were active in our glaucoma model. F-VEP results showed that the visual function of mice decreased gradually after intracameral injection of the cross-linking hydrogel. The latent period of F-VEP in mice with COH was longer, and the amplitude was reduced compared with that in the control group. Our model shows a chronic neurodegeneration in RGCs and significant loss of optic nerve axons. The positive rate of the RGC TUNEL assay in mice with COH was significantly increased compared with that in control mice.

As a biocompatible and multifunctional material, the cross-linked hydrogels have presented flexible characteristics for various applications in the clinical practice of ophthalmology, including ocular surface treatment, contact lenses of drug delivery systems for glaucoma treatment, and substitutes for vitreous (20–22). Cross-linking hydrogels, such as thiol-modified hyaluronic acid, have the advantage of *in situ* gelling by premix-separating components in different proportions at appropriate time points to control gelation (23). In addition, to induce the IOP elevation successfully, the cross-linking hydrogel should be able to gel rapidly immediately after intracameral injection or be diluted by the aqueous humor. Our study showed that the IOP elevation was induced by injection of the cross-linking hydrogel at a ratio of 4:1 and the cross-linking hydrogel gel *in situ* for ~5 min. The elevated IOP is stable, and a high success rate of induction of IOP elevation is observed (the success rate is 68%). Hydrogels can be retained in the eye for a long time due to their good biocompatibility and non-degradable properties. Hyaluronic acid (HA) is the main component of cross-linking hydrogels, and it is highly biocompatible. *In vitro* studies have shown that *in situ* gelation and gel incubation of HA are not toxic to eye cells. *In vivo* studies have shown that *in situ* cross-linked hydrogels injected into the eyes of non-human primates and rats have good biocompatibility and no obvious adverse reactions (24–26). The mechanism of COH induced by intracameral injection of cross-linking hydrogel is the blocking of the outflow pathway of aqueous humor. The gel droplets formed by injecting the cross-linking hydrogel into the anterior chamber exhibit a physical blocking effect, which can prevent the outflow pathway of aqueous humor and achieve long-term stable maintenance of COH. In terms of operation, we used a corneal long tunnel incision that can self-seal after surgery. In addition, after the cross-linked hydrogel was completely injected into the anterior chamber, the syringe was stopped *in situ* for 5 min, and the syringe was pulled out after *in situ* cross-linked gelation to further prevent liquid leakage and ensure the effect of increasing and maintaining the intraocular pressure of our model.

Many types of animal models for IOP elevation have been described. In some models, glaucoma is caused by blocking outflow of aqueous humor, including injection of microbeads and hyaluronic acid into the anterior chamber, injection of hypertonic saline into the episcleral veins or limbal vessels, occlusion of the episcleral veins by cauterization or suture ligation, and laser photocoagulation of the trabecular meshwork at the limbus (9–11, 27). In addition, DBA/2J mice have also been studied as glaucoma models. However, in existing models, there have always been technical challenges in maintaining a clear optical media to facilitate the study of the structural and functional integrity of RGCs. For laser photocoagulation and episcleral vein cauterization or ligation, maintaining the clarity of the cornea is typically not easy to achieve (12). Laser photocoagulation typically requires 60–80 photocoagulated spots on the trabecular meshwork and scleral surface veins of the mouse eye, which is very difficult and requires repeated operations. Due to the small size of the mouse eye, it requires high technical requirements for the operator and special laser equipment (28). The success rate of laser photocoagulation-induced COH ranged from 80 to 90% for a single laser treatment lasting for 4 to 8 weeks (29, 30). Superior scleral vein burning or ligation can significantly increase IOP, and the mechanism may be caused by congestion in the uveal vascular system. This method is simple to perform. However, the method also blocks the outflow of blood in the eye, resulting in congestion and ischemic changes in the eye, which makes the injury factors more complicated. The blood return flow in the unoccluded superior scleral vein in the experimental eye may be increased to compensate, and the phenomena of occluded paravenous angiogenesis and recanalization are also noted. These changes may lead to a decrease in IOP, resulting in the failure of the model and a low success rate. Zhao et al. (31) reported the success rate was 50%, and the duration of IOP elevation was 12 weeks. The procedure has a high rate of complications. Hyphema occurred in 71% of the mice, and suture breaks occurred in 29% of the mice (31). For the intracameral injection of microbeads, a second injection is always required, which means an additional invasive operation and possible side effects (such as inflammation and infection) (32). It was reported that a single injection of microbeads resulted in a 30% success rate in elevating IOP and persisted for more than 3 weeks (27). Intracameral injection of hyaluronic acid requires repeated injection to maintain a high IOP because hyaluronic acid is easily metabolized, degraded, and absorbed in the eye. Thus, there was local corneal edema and severe inflammation around the injection site. A single injection of elevated IOP lasted for up to 7 days (33). In DBA/2J mice, IOP began to spontaneously increase from 7 to 8 months after birth. The increase in IOP was due to mutations in two genes GPNMB and TyrPL in mice. These mutations led to the depigmentation of the iris, and the shed pigment and cell debris blocked the outflow of atrial fluid, thus causing high IOP. By 10 to 12 months after birth, IOP begins to decrease again due to lesions in the ciliary epithelial cells. However, the DBA/2J experimental glaucoma model showed variability in expression, and 22% of the animals developed major systemic complications (34). This feature makes it difficult to assess disease progression and study the structure and function of RGCs in glaucoma. Episcleral vein injection of hypertonic saline can cause the sclerosis of the aqueous humor outflow system in mice, which increases the resistance of aqueous humor outflow and leads to an increase in IOP. Kipfer-Kauer et al. (35) reported that in the case of reintervention, the success rate was 100% and the elevated IOP was sustained for 6 weeks. However, this model is difficult to operate, and a special microsyringe equipment is needed, which requires higher operating skills for the operator. Chan et al. (12) reported the use of a homemade cross-linking hydrogel for intracameral injection to induce COH in mice. The daily mean IOP ranged between 14 ± 3 and 24 ± 3 mmHg, which was similar to our study. They reported that the elevated IOP was sustained for 4 weeks. In our study, our model preserves the clarity of optical media. The IOP elevation induced by the cross-linking hydrogel could be sustained for at least 8 weeks with only one injection, which helped to avoid the risk of inflammatory responses caused by additional operations. In addition, we used commercial hydrogels to ensure the stability of the hydrogel products. Therefore, the model has strong operability.

In recent years, increasing attention has been given to the role of the immune inflammatory response in glaucoma optic nerve damage (36). Retinal microglial cells are one of the main cells involved in the immune inflammatory response in the retina and optic nerve. During retinal and optic nerve injury, microglial cells play a neuroprotective role by morphologically changing, proliferating, and migrating to the damaged site to phagocytose and eliminate microbes, protein aggregates, and dead cells (6). However, excessive activation of microglial cells leads to damage of the nerve tissue by releasing a series of toxic substances (such as TNF-α, IL-1β, etc.) (6). Studies have shown that microglial cells are involved in the pathological process of glaucoma. Neufeld (37) found that microglial cells were activated in the optic papilla of glaucoma patients, and cell morphology and distribution were changed. Wang et al. (38) found that in an animal model of glaucoma, activated microglial cells appeared in the RGC layer only 2 h after IOP elevation. CD68 is an activation marker of microglial cells. In our study, CD68 expression was observed after 2 weeks of COH and microglial cells have a transition from slender branching to globular amoeboid morphology. The number of microglial cells and Iba-1 and CD68 protein levels increased in COH mice over time. Some studies have analyzed the microglial activation in glaucoma models in mice at different times after ocular hypertension induction. CD68 and MHC-II expression were observed in the nerve fiber layer–ganglion cell layer after 15 days of unilateral laser-induced experimental glaucoma model (13). These findings were similar to those in our study. Others reported that the number of Iba-1+ microglial cells was increased in laser-induced ocular hypertension eyes compared with control eyes at 3, 5, 8, and 15 days, and the peak number of Iba-1+ microglial cells occurred at 3 days (39).

IL-17A is a cytokine that is secreted mainly by activated CD4+ T cells (Th17 cells). The IL-17 family is composed of six structurally similar cytokines (IL-17A~IL-17F) and five receptors (IL-17RA~IL-17RE) (40). These cytokines are dimeric molecules with sizes of 23~36 kDa composed of 163~202 amino acids (40). IL-17a, a well-studied cytokine in the IL-17 family, plays an important role in the immune inflammatory response, angiogenesis, and the occurrence and development of tumors (41). Studies have shown that IL-17A is involved in the pathogenesis of CNS neurodegenerative diseases. In recent years, some researchers have studied IL-17A levels in patients with glaucoma. The frequency of IL-17A-secreting cells and IL-17A+ CD4 T cells is significantly higher in patients with glaucoma compared with controls (42). In our study, the protein levels of IL17, TNF-α, and IL-1β were upregulated from 2 to 4 weeks postelevated IOP and then further increased at 6 weeks postelevated IOP. Various cells have been reported to produce IL-17A, one of which is microglial cells (43). In our model, IL-17A, TNF-α, and IL-1β may be produced by activated microglial cells during COH. Elevated IL-17A and retinal nerve injury caused by COH further could activate microglial cells and astrocytes and could transform microglia into an M1- or M2-like phenotype and astrocytes into an A1- or A2-type phenotype, respectively. Microglial cells often undergo a dynamic process during injury, which is characterized by the mixing or conversion between the M1 and M2 phenotype (44). Classical M1-like microglial cells produce high levels of proinflammatory cytokines (such as TNF-α and IL-1β). In addition, activated astrocytes and microglial cells recruit monocytes, macrophages, and T cells to cross the blood–eye barrier into the retina and increase the secretion of proinflammatory cytokines. Elevated levels of TNF-α, IL-1β, and IL-17A further stimulate microglia and astrocytes and increase their activity. Proinflammatory cytokines and glial cells in the retina form a tight positive feedback loop. As the disease progresses, the levels of proinflammaroy cytokines increase (45). To our knowledge, we are the first to report increased IL-17A expression in an experimental glaucoma model. Further research is needed.

VEP refers to the cortical electrical activity recorded after visual stimulation. The electrophysiological signal is generated in the striatum and extrastriate cortex (46). VEP provides a useful tool for assessing the functional retina and cortex and the state of visual pathways from the retina to the cortex. In an animal model of glaucoma, alteration of the inner retinal circuitry was found to precede RGC degeneration and optic nerve atrophy (46). Thus, VEP contributes to characterizing the progression of glaucoma. In DBA/2J mice with ocular hypertension, the amplitude of VEP was clearly reduced (47). In our study, the amplitude and latent period of mice at 2, 4, and 6 weeks of COH were significantly different compared with those in the control group.

In acute IOP elevation, RGC loss was induced in the first week of injury but not thereafter (48). In an animal model of laser photocoagulation-induced intraocular hypertension, the rate of RGC loss was 20–30% at 8 weeks (49) and the rate of axon loss was 59% at 24 weeks (50). Zhu et al. (51) reported that the rate of RGC loss was 30% at 11 weeks and 8% at 12 weeks by Zhao et al. (31) in the model of episcleral vein cauterization and circumlimbal suture. Chan et al. (12) reported that the survival rate of RGCs was 37% in mice at 4 weeks of COH. In our study, we found that RGCs and optic nerve axons were gradually lost over time in the eyes after IOP elevation. The rates of RGC loss were 46.8% in 1/6 retinal radius, 39.2% in 3/6 retinal radius, and 45.1% in 5/6 retinal radius at 8 weeks of COH, respectively. The rate of optic nerve axon loss was 69.5% at 6 weeks of COH. In different animal models of ocular hypertension, the number of RGC in control mice was significantly different. Liu et al. (52) reported that in the model of circumlimbal suture, the number of RGC was 3,098 ± 189/mm^2^ (×20 magnification). However, in the same animal model by Zhu et al. (51), the number of RGC was approximately 720/mm^2^ in peripheral retina (×200 magnification). In our model, the number of RGCs was 1,369.0 ± 77.4/mm^2^ in 1/6 retinal radius, 1,046.0 ± 175.3/mm^2^ in 3/6 retinal radius, and 795.0 ± 43.8/mm^2^ in 5/6 retinal radius of the control group, respectively (×200 magnification). The difference in the number of RGC may be caused by different magnifications. Together, these results support that intracameral injection of cross-linking hydrogel damaged the inner retina and is an efficient model to study the functional degeneration of the RGCs.

In summary, we established a new COH model induced by intracameral injection of the cross-linking hydrogel. The model worked efficiently to demonstrate the features simulating glaucoma. Therefore, we provide a new, effective, and simple animal model for glaucoma research.

## Data Availability Statement

The raw data supporting the conclusions of this article will be made available by the authors, without undue reservation.

## Ethics Statement

The animal study was reviewed and approved by Ruijin Hospital, Shanghai Jiao Tong University School of Medicine Animal Care and Use Committee.

## Author Contributions

JC wrote and edited the manuscript. JS provided pictures of some of the results. HY, PH, and YZ edited the manuscript. All authors read and approved the final manuscript.

## Conflict of Interest

The authors declare that the research was conducted in the absence of any commercial or financial relationships that could be construed as a potential conflict of interest.
