# Corrigendum: Evaluation of the Effectiveness of a Chronic Ocular Hypertension Mouse Model Induced by Intracameral Injection of Cross-Linking Hydrogel

**DOI:** 10.3389/fmed.2021.687339

**Published:** 2021-04-30

**Authors:** Junjue Chen, Jun Sun, Huan Yu, Ping Huang, Yisheng Zhong

**Affiliations:** ^1^Department of Ophthalmology, Ruijin Hospital, Shanghai Jiao Tong University School of Medicine, Shanghai, China; ^2^Shanghai Key Laboratory for Bone and Joint Diseases, Shanghai Institute of Traumatology and Orthopedics, Ruijin Hospital Affiliated Medical School, Shanghai Jiaotong University, Shanghai, China

**Keywords:** glaucoma, animal model, mice, cross-linking hydrogel, intracameral injection

In the original article, there was an error. The corresponding author's email was incorrectly spelled as “yszhong@126.com.” The correct email is yszhong68@126.com.

The authors apologize for this error and state that this does not change the scientific conclusions of the article in any way. The original article has been updated.

